# Change in Fish Community Structure in the Barents Sea

**DOI:** 10.1371/journal.pone.0062748

**Published:** 2013-04-29

**Authors:** Michaela Aschan, Maria Fossheim, Michael Greenacre, Raul Primicerio

**Affiliations:** 1 Faculty of Biosciences, Fisheries and Economics, University of Tromsø, Tromsø, Norway; 2 Institute of Marine Research, Tromsø, Norway; 3 Department of Economics and Business, Universitat Pompeu Fabra, Barcelona, Spain; Technical University of Denmark, Denmark

## Abstract

Change in oceanographic conditions causes structural alterations in marine fish communities, but this effect may go undetected as most monitoring programs until recently mainly have focused on oceanography and commercial species rather than on whole ecosystems. In this paper, the objective is to describe the spatial and temporal changes in the Barents Sea fish community in the period 1992–2004 while taking into consideration the observed abundance and biodiversity patterns for all 82 observed fish species. We found that the spatial structure of the Barents Sea fish community was determined by abiotic factors such as temperature and depth. The observed species clustered into a deep assemblage, a warm water southern assemblage, both associated with Atlantic water, and a cold water north-eastern assemblage associated with mixed water. The latitude of the cold water NE and warm water S assemblages varied from year to year, but no obvious northward migration was observed over time. In the period 1996–1999 we observed a significant reduction in total fish biomass, abundance, mean fish weight, and a change in community structure including an increase in the pelagic/demersal ratio. This change in community structure is probably due to extremely cold conditions in 1996 impacting on a fish community exposed to historically high fishing rates. After 1999 the fish community variables such as biomass, abundance, mean weight, P/D ratio as well as community composition did not return to levels of the early 90s, although fishing pressure and climatic conditions returned to earlier levels.

## Introduction

The Barents Sea ecosystem has been considered ecologically ‘healthy’ [1], [2] and many of the commercial fish stocks, especially the Northeast Arctic cod (*Gadus morhua*), are in good shape [3]. However, due to rapid climate change in the Arctic, where local temperature increase is expected to be twice the global average [4], [5], major changes in the marine ecosystems are expected. In the adjacent North Sea, changes in large-scale hydro-meteorological forcing has caused a change in individual species and key ecosystem parameters, such as diversity from phytoplankton to fish [6]. Local temperature increase in the Barents Sea is expected to lead to migration of Atlantic fish species northwards [5], [7], [8], but more complex and structural community changes may also occur [9]–[11].

The Barents Sea has been studied for several decades, and approximately 500 survey days are spent in the vast sea area annually. Change in climatic conditions can cause structural alterations in marine fish communities, but this effect may go undetected as the monitoring programs in the Barents Sea have, until 2004 [12], mainly focused on oceanography and commercial species (e.g. 0-group surveys on commercial juveniles, shrimp surveys and gadoid fish surveys) rather than on the whole ecosystem. Single species responses may not give a good indication of possible changes in the ecosystem due to the large inter-annual variability of single stocks, and because fishing may conceal climate change effects [13]. In general, change in climatic conditions and fishing have been the most important drivers for structural change in marine ecosystems [14], for instance as seen in the previously cod-dominated community in the NW-Atlantic [15].

A community change may consist of a change in structural properties, such as species composition or change in functional roles. Changes in species composition can be indicative of ecological regime shifts [16], [17]. The evidence for such shifts in the oceans, for instance a persistent change in biomass or structural changes over several trophic levels, has been documented for the North Pacific [18], [19], the Northwest Atlantic [15], and the North Sea [6]. Such ecological changes are often related to shifts in oceanographic conditions and overfishing [10], and may be difficult to reverse. Regime shifts have only been detected through retrospective analysis [20], [21], for instance through benthic-pelagic decoupling in Northwest Atlantic fish communities resulting in increased pelagic fish abundance and biomass [22]–[24]. Pelagic species change abundance and distributions more rapidly than demersal species, due to faster lifecycles and smaller body sizes [25], [26], while both may be subject to fishing. The pelagic/demersal (P/D) ratio of the fish community is therefore a suitable descriptor of temporal changes in an ecosystem.

When investigating community change, the baseline community structure needs to be known. For the Barents Sea, the fish fauna and the biogeographical distribution patterns of species are well known from extensive taxonomical studies [27]. Fish community studies, i.e. descriptions of assemblages of species inhabiting specific habitats or sub-areas of the Barents Sea, are limited in geographical scope and time 28–34. These studies show that assemblages can be identified in subareas, but they do not provide information on temporal variability.

The main objective of this study is to document the temporal and spatial changes in the Barents Sea fish assemblages in relation to environmental parameters. Fish species that are not targeted by fisheries are included in the analysis, providing valuable, additional ecological information on structural changes [35], [36]. Spatial and temporal changes in the Barents Sea fish communities are investigated by studying fish biomass, abundance, mean weight, diversity, P/D ratio and species composition in relation to driving forces, such as bottom temperature, the North Atlantic Oscillation (NAO) index and fishing (demersal landings).

## Materials and Methods

### Ethics Statement

The surveys were conducted by marine research institutes financed by the Norwegian Government and approval for trawling was given by the Directorate of Fisheries. No specific ethical approval was applied for 1992–2004 as this was not required at that stage. Some of the species caught are little known and the deep water redfish (*Sebastes mentella*) is red listed (http://www.iucnredlist.org/). The surveys have contributed to additional knowledge about these species and also provided information for area closures to protect juveniles of red fish for example.

### Field Study

**Figure 1:**
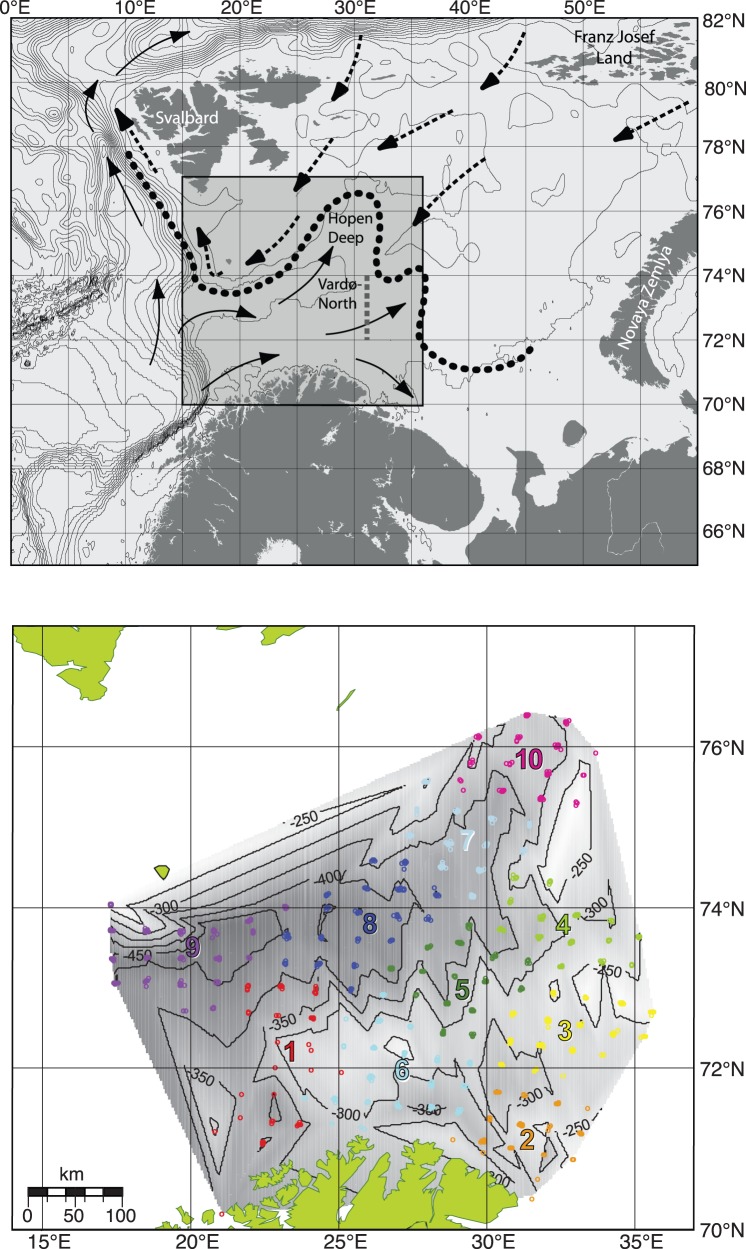
Main surface currents, bathymetry and sampling regions in the Barents Sea. (*a*) Atlantic currents (**–>**) and Arctic currents (**–>**) and the mean position of where these water masses meet, at the depth of 20–100 m, the Polar Front (**• • •**) that most years follows the 200 m depth isoline that limits the study area in the North. The Vardø-North section located at 31°13′E. The study area is indicated by the grey square. In (*b*) bathymetry (shaded with isolines) based on information from all stations in 1992−2004 and stations (dots) with color of region (R1–R10). Stations shallower than 200 m have been excluded in all years.

Data on fish species abundance and biomass for 82 fish taxa were collected during the former annual shrimp surveys conducted by the Norwegian Institute of Fisheries and Aquaculture (NIFA) and the Institute of Marine Research (IMR) in the Barents Sea from 1992 until 2004, when the shrimp survey was terminated. Survey year, vessel used, institution in charge, departure date, number of survey days and number of stations sampled are presented in Table 1. The study area ranged from 70°N to 77°N and from 15°E to 36°E, and the depth at stations sampled varied between 100 m and 495 m (Fig. 1a). Stations were placed on a grid with 20−30 nautical miles distance between stations [37].

**Table 1:** Survey year, vessel used, institution in charge (NIFA: Norwegian Institute of Fisheries and Aquaculture, IMR: Institute of Marine Research), departure date, number of survey days and number of stations sampled.

Year	Institute	Vessel	Dep. Date	Nr. days	Nr.stations
1992	NIFA	M/T Gargia	02. May	29	176
1993	NIFA	R/V Jan Mayen	22. April	20	141
1994	NIFA	R/V Jan Mayen	25. April	22	112
1995	NIFA	R/V Jan Mayen	18. April	20	125
1996	NIFA	R/V Jan Mayen	15. April	20	141
1997	NIFA	R/V Jan Mayen	19. April	22	91
1998	NIFA	R/V Jan Mayen	19. April	18	110
1999	NIFA	R/V Jan Mayen	15. April	15	97
2000	NIFA	R/V Jan Mayen	18. April	19	123
2001	NIFA	R/V Jan Mayen	21. April	15	90
2002	IMR	R/V Jan Mayen	16. April	17	107
2003	IMR	R/V Jan Mayen	14. April	21	109
2004	IMR	R/V Jan Mayen	12. April	21	127

A total of 1549 stations were sampled in the 13 years of shrimp surveys conducted. The station spacing and number of stations changed between years (Table 1), so that the area covered in all years was roughly the same. In order to take into account the spatial nature of the samples in the multivariate analysis, the sampling area was partitioned into 10 regions based on a k-means clustering algorithm of the distances between stations (Fig. 1b). The survey trawl (Campelen 1800), a shrimp trawl by design, is widely used in ground fish surveys (e.g. in the Barents Sea, the North Sea and off Newfoundland), and has a good catchability for demersal fish [38]. However, several species with more pelagic characteristics are also regularly caught in this trawl. The mean depth was recorded for each haul. A temperature sensor (Scanmar) was attached to the head-rope of the survey trawl to ensure a bottom temperature estimate at each station. The temperature sensor was calibrated against CTD measurements.

During the annual shrimp survey all fish were identified, counted, and total weight per species recorded. The dataset was standardized to ensure that sampling effort did not bias results between years. The 1992 station grid was used as reference. Stations closest to reference were chosen for each year, then replicate samples at stations in a year as well as stations shallower than 200 m were excluded (189 in total out of the 1549 leaving 1360 stations). There were imbalances between the 10 regions in terms of number of samples taken from year to year, and this could lead to finding inter-annual differences that are due to these sampling imbalances rather than real changes. To avoid this sampling bias, the data (abundance by species, mean weight, biomass and bottom temperature) from each station were reweighted to reflect equal representation in each region across the years. For example, in 1992 region 1 in the south-west had 17 samples whereas for all other years there were no more than 7, in some cases only one sample – the abundances in region 1 were thus down-weighted in 1992, and the under sampled regions similarly up-weighted to reflect the same level of sampling in all years.

### Data Analysis

All statistical analyses were run with the software R 2.10.0 [39], using the R package vegan [40] for multivariate analyses. After the reweighting of the stations, described above, to correct for sampling disparity between years, the biomass, abundance, species number, mean weight over all species and size classes, and the Shannon–Weaver diversity index (*H′*) in each haul was calculated as:
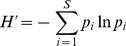
(1)where *p_i_* is the proportion of total abundance of species *i* and *S* is the number of species [41].

The P/D ratio was calculated from the data set as the sum of pelagic fish abundance divided by the sum of demersal fish abundance [24], [42] as a coarse metric of fish community structure. Information for the classification of species habitat was obtained from FishBase [43] and was used to define the demersal group and extend the pelagic group to include largely planktivorous species that are demersal in habit, but during night leave the bottom to feed on plankton. Species characterized as bathydemersal (habitat category “P-D” in Table 2) were excluded, and when generic names did not allow for separation between species with different habitat preference (e.g. Sebastes spp.), the species group was excluded from the P/D ratio calculation (Table 2).

**Table 2:** Taxa identified in the SW Barents Sea in spring 1992−2004.

Scientific name	Abbreviation		Common name	Habitat			% ofind.		% ofstations		Yearspresent	
PANDALIDAE												
*Pandalus borealis* (Krøyer, 1838)			Shrimp						94.3		13	
CHIMAREIDAE												
*Chimaera monstrosa* Linnaeus, 1758			Rabbit fish				+		+		1	
DQUALIDAE												
*Somniosus microcephalus* (Bloch & Shneider, 1801)			Greenland shark				+		+		1	
RAJIDAE												
*Amblyraja hyperborea* (Collett, 1879)	Ra spp		Arctic skate	D			+		0.9		7	
*Amblyraja radiata* (Donovan, 1808)	Ra spp		Thorny skate	D			0.6		64.6		13	
*Bathyraja spinicauda* (Jensen, 1914)	Ra spp		Spinetail ray	D			+		1.5		9	
*Dipturus batis* (Linnaeus, 1758)	Ra spp		Blue skate	D			+		+		7	
*Raja clavata* Linnaeus, 1758	Ra spp		Thornback ray	D			+		2.1		10	
*Rajella fyllae* (Lütken, 1887)	Ra spp		Round ray	D			+		1.7		10	
*Rajidae spp*	Ra spp		Skates	D			+		3.9		4	
CLUPEIDAE												
*Clupea harengus* Linnaeus, 1758	Cl ha		Herring	P			0.8		24.3		13	
OSMERIDAE												
*Mallotus villosus* (Müller, 1776)	Ma vi		Capelin	P			9.0		68.8		13	
ARGENTINIDAE												
*Argentina silus* (Ascanius, 1775)	**Ar si**		Greater argentine	P			+		2.6		12	
*Argentina sphyraena* Linnaeus, 1758			Lesser argentine				+		0.5		4	
STENOPYCHIDAE												
*Maurolicus muelleri* (Gmelin, 1789)	Ma mu		Pearlsides				+		0.3		4	
MYCTOPHIDAE												
*Benthosema glaciale* (Reinhardt, 1837)	Be gl		Glacier lanternfish	P			+		3.6		11	
PARALEPIDAE												
*Arctozenus risso* (Bonaparte, 1840)	Ar ri		Ribbon barracudina	P			0.2		16.9		12	
GADIDAE: GADINAE												
*Boreogadus saida* (Lepetchin, 1774)	Bo sa		Polar cod	P			4.1		26.0		13	
*Gadiculus argenteus thori* Schmidt, 1914	Ga at		Silvery pout	D			+		+		7	
*Gadus morhua* Linnaeus, 1758	Ga mo		Cod	D			17.1		95.3		13	
*Melanogrammus aeglefinus* (Linnaeus, 1766)	Me ae		Haddock	D			9.5		71.9		13	
*Merlangius merlangus* (Linnaeus, 1758)			Whiting				+		+		2	
*Micromesistius poutassou* (Risso, 1827)	Mi po		Blue whiting	P			2.0		26.4		13	
*Pollachius virens* (Linnaeus, 1758)	**Po vi**		Saithe	D			+		6.4		12	
*Trisopterus esmarkii* (Nilsson, 1855)	**Tr es**		Norway pout	P-D			1.9		24.4		13	
GADIDAE: LOTINAE												
*Brosme brosme* (Ascanius, 1775)	Br br		Tusk	D			+		1.2		10	
*Ciliata mustela* (Linnaeus, 1758)			Five-bearded rockling				+		0.1		3	
*Enchelyopus cimbrius* (Linnaeus, 1766)	Ga spp		Four-bearded rockling	D			+		2.4		10	
*Gaidropsarus argentatus* (Reinhardt, 1937)	Ga spp		Arctic rockling				+		0.2		1	
*Gaidropsarus vulgaris* (Cloquet, 1824)	Ga spp		Three-bearded rockling	D			+		0.9		4	
*Molva dipterygia* (Pennant, 1784)	Mo di		Blue ling				+		0.1		2	
*Molva molva* (Linnaeus, 1758)	Mo mo		Ling				+		+		1	
*Raniceps raninus* (Linnaeus, 1758)	Ra ra		Tadpole fish				+		+		1	
MACROURIDAE												
*Macrourus berglax* Lacepéde, 1801	Ma be		Onion-eye grenadier	D			+		4.3		12	
ZOARCIDAE												
*Gymnelus retrodorsalis* Le Danois, 1913	Ly spp		Aurora unernak	D			+		0.4		3	
*Lycenchelys kolthoffi* Jensen, 1904	Ly spp		Checkered wolf eel	D			+		0.5		5	
*Lycenchelys sarsii* (Collet, 1871)	Ly spp		Sars’ wolf eel	D			+		+		1	
*Lycodes esmarkii* Collet, 1875	**Ly es**		Greater eelpout	D			0.2		13.7		12	
*Lycodes eudipleurostictus* Jensen, 1902	Ly eu		Doubleline eelpout	D			+		7.7		12	
*Lycodes frigidus* Collet, 1879	Ly spp		Glacial eelpout	D			+		0.4		4	
*Lycodes gracilis* M. Sars, 1867*	Ly gr		Vahl’s eelpout	D			0.7		53.4		13	
*Lycodes pallidus* Collet, 1879	Ly spp		Pale eelpout	D			+		1.0		4	
*Lycodes reticulatus* Reinhardt, 1835	Ly spp	Arctic eelpout			D	+		1.5		4		
*Lycodes rossi* Malmgren, 1865	Ly spp	Threespot eelpout			D	+		7.7		8		
*Lycodes seminudus* Reinhardt, 1937	Ly spp	Longear eelpout			D	+		1.0		5		
*Lycodes spp*	Ly spp	Eelpout (spp.)			D	+		6.1		6		
*Lycodes squamiventer* Jensen, 1904	Ly spp	Scalebelly pout			D	+		+		1		
*Lycodonus flagellicauda* (Jensen, 1902)	Ly spp	Eelpout sp. 1			D	+		+		2		
SCORPAENIDAE												
*Sebastes marinus* (Linnaeus, 1758)	Se spp	Golden redfish				2.0		25.8		13		
*Sebastes mentella* (Travin, 1951)	Se spp	Beaked redfish				23.1		71.9		12		
*Sebastes viviparus* Krøyer, 1845	Se spp	Norway redfish				+		0.8		4		
*Sebastes spp*	Se spp	Redfish (spp.)				6.9		20.5		8		
GASTEROSTERIDAE												
*Gasterosteus aculeatus aculeatus* (Linnaeus, 1758)	Ga aa	Three-spines stickleback			P	+		0.8		4		
COTTIDAE												
*Artediellus atlanticus* Jordan & Evermann, 1898	**Ar at**	Atlantic hookear sculpin			D	0.9		41.5		13		
*Myoxocephalus scorpius* (Linnaeus, 1758)	My sc	Shorthorn sculpin			D	+		1.1		7		
*Triglops murrayi* Günther, 1888	Tr spp	Moustache sculpin			D	0.2		8.1		13		
*Triglops pingelii* Reinhardt, 1837	Tr spp	Ribbed sculpin			D	+		0.9		2		
*Triglops spp*	Tr spp	Triglops sculpins			D	0.1		4.5		9		
COTTINCULIDAE												
*Cottunculus microps* Collet, 1875	Co mi	Polar sculpin			D	+		3.9		12		
AGONIDAE												
*Agonus cataphractus* (Linnaeus, 1758)	Ag ca	Hook nose				+		+		2		
*Leptagonus decagonus* (Bloch & Schneider, 1801)	**Le de**	Atlantic poacher			D	0.5		29.3		13		
*Ulcina olrikii* (Lytken, 1877)		Arctic alligatorfish				+		+		1		
CYCLOPTERIDAE												
*Cyclopterus lumpus* Linnaeus, 1758	Cy lu	Lumpsucker			P	+		13.1		13		
*Eumicrotremus spinosus* (Fabricius, 1776)		Atlantic spiny lumpsucker				+		0.3		4		
LIPARIDAE												
Careproctus sp**	Ca sp	Sea snail sp. 1			P-D	0.1		26.2		13		
*Liparis bathybii* (Collet, 1879)	Li ba	Black seasnail				+		0.3		4		
*Liparis fabricii* Krøyer 1847	Li fa	Gelatinous seasnail			P-D	+		3.3		10		
*Liparis gibbus* Bean, 1881***	Li gi	Variegated snailfish			D	+		1.7		3		
*Liparidae spp*	Li spp	Snailfishes (spp.)				+		0.4		3		
STICHAEIDAE												
*Anisarchus medius* (Reinhardt, 1837)	An me	Stout eelblenny				+		0.4		3		
*Leptoclinus maculatus* (Fries, 1837)	**Le ma**	Spotted snake blenny			D	0.3		13.3		13		
*Lumpenus lampraetaeformis* (Walbaum,1972)	**Lu la**	Snake blenny			D	+		9.5		12		
*Stichaeidae spp*	St spp	Pricklebacks				+		0.3		2		
ANARHICHADIDAE												
*Anarhichas denticulatus* Krøyer, 1845	**An de**	Northern wolffish			D	0.2		39.9		13		
*Anarhichas lupus* Linnaeus, 1758	An lu	Atlantic wolffish			D	+		3.7		11		
*Anarhichas minor* Olafsen, 1772	An mi	Spotted wolffish			D	+		18.2		13		
PLEURONECTIDAE												
*Glyptocephalus cynoglossus* (Linnaeus, 1758)	Gl cy	Witch flounder				+		0.9		8		
*Hippoglossoides platessoides* (Fabricius, 1780)	Hi pl	Long rough dab			D	17.9		98.5		13		
*Hippoglossus hippoglossus* (Linnaeus, 1758)	Hi hi	Halibut				+		0.1		1		
*Limanda limanda* (Linnaeus, 1758)		Dab				+		+		1		
*Microstomus kitt* (Walbaum, 1792)	Mi ki	Lemon sole				+		0.1		1		
*Pleuronectes platessa* Linnaeus, 1758	Pl pl	European plaice				+		0.4		4		
*Reinhardtius hippoglossoides* (Walbaum, 1792)	**Re hi**	Greenland halibut			P-D	0.8		60.8		13		

Abbreviations are given for all taxa included in the CCA, and indicator species for the three assemblages in bold. Habitat is indicated as pelagic (P), demersal (D) or pelagic-demersal (P-D).

Percentages <0.1% denoted by +.

*Lycodes gracilis* M. Sars, 1867* eq *Lycodes vahlii* Reinhardt, 1831.

Careproctus** sp eq *Careproctus derjugini* Chernova 2005 and eq *Careproctus reinhardthi* (Krøyer,1862).

*Liparis gibbus* Bean, 1881*** eq *Liparis liparis* (Linnaeus, 1766).

The structural variation of the fish community abundance in space and time was modeled as a function of region, depth, bottom temperature and year by direct ordination, using canonical correspondence analysis (CCA). Due to some inconsistencies in identification it was appropriate to group species for the ordination analysis; the redfish *Sebastes mentella*, *S. marinus*, *S. viviparous* and *S.* spp. were all treated as one variable (Se_spp). All *Rajidae* (Ra_spp), all *Triglops* (Tr_spp) and some of the *Lycodes* (Ly_spp) were treated as one taxon respectively (Table 2). In addition, taxa occurring only once in the whole data set where excluded.

To unify the interpretation of the results, all environmental variables were coded as categorical variables. The discrete variable year was categorized as a set of 13 “crisp” dummy (zero/one) variables, one for each year, while continuous variables such as depth and bottom temperature were each coded into four “fuzzy” dummy variables adding up to 1, using so-called triangular membership functions [44]. This coding scheme loses no information in the data and has the advantage that nonlinear relationships can be diagnosed. The four so-called “hinge points” used in the creation of the fuzzy categories were: for depth, 206 (minimum depth), 300, 400 and 495 meters (maximum depth), and for bottom temperature.

–1.4 (minimum temperature), 1, 3 and 6.4 degrees Celsius (maximum temperature). Regional group was also coded into dummy variables in a fuzzy way so that the coding of stations on the borderline between two regions would be coded 0.5 and 0.5, for example, for the respective regions, whereas a station near the center of a region would be coded totally (1) into that region – like the coding of depth and temperature, this strategy conserves the maximum information about the geographical locations of the stations. The CCA model was tested by Monte Carlo permutation [40], [45].

Previously identified fish assemblages in the Barents Sea [30] are the cold water NE assemblage, with four key species (*Artediellus atlanticus, Leptagonus decagonus*, *Leptoclinus maculates* and *Lumpenus lampraetaeformis*) and the warm water S assemblage with three key species (*Argentina silus*, *Pollachius virens* and *Trisopterus esmarkii*). We have identified the stations where at least a total of 10 individuals of the key species of the respective assemblage were observed. Maps with S assemblage and NE assemblage stations were drawn for each year. The annual distributions of the warm water S assemblage and the cold water NE assemblage, in the period 1992 to 2004, were then integrated in one map.

## Results

### Temporal Change in Biomass, Abundance, Diversity and P/D-ratio

**Figure 2:**
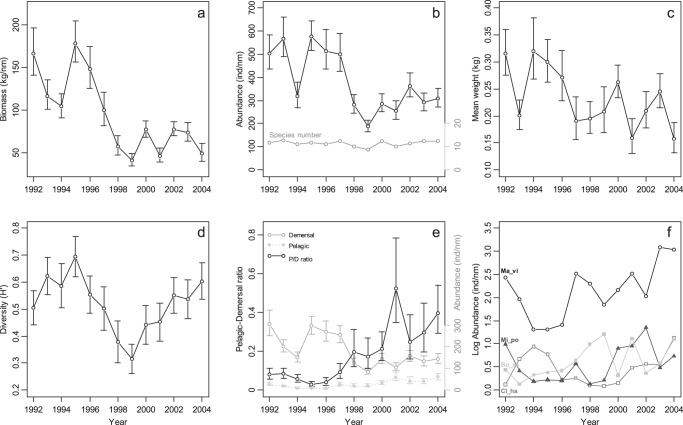
Fish community parameters for each year 1992−2004. The mean biomass (*a*), mean abundance and (with scale on right) species number (*b*), mean weight of individual fish (*c*), Shannon–Weaver diversity (*H’*) (*d*), the mean P/D ratio and (with scale in the right) pelagic and demersal fish abundances (*e*), and mean log-transformed abundance of four pelagic species (*f*) *Mallotus villosus* (circles), *Boreogadus saida* (solid gray squares), *Micromesistius poutassou* (triangles) and *Clupea harengus* (open squares). The 95% confidence intervals for the means are shown in most cases based on the log-transformed data after reweighting to be representative of the sampling regions.

The biomass, abundance and the annual mean weight of individual fish decreased from 1996 to 1999, then increased, but remained at a lower level than prior to 1996 (Fig. 2a–c). The Shannon-Weaver diversity (*H′*) also declined from 1996 to 1999 but then increased again (Fig. 2d). The overall mean species number is 11.5, yet in 1999 it dropped to 8.7 (Fig. 2b), and contributes to the decline in diversity in the same year (Fig. 2d).

The P/D ratio increased (Fig. 2e), revealing that the fish community has become more dominated by small pelagics after 1996. The abundance of demersal fish including dominant species belonging to e.g. *Cottidae*, *Rajidae*, and *Gadidae*, (*Gadus morhua* and *Melanogrammus aeglefinus*) decreased (Fig. 2e), while that of pelagic species such as *Mallotus villosus*, *Boreogadus saida, Micromesistius poutassou* and *Clupea harengus* increased (Fig. 2e, 2f).

### Spatial Patterns and Temporal Change in Community Structure

The CCA allows stations and species to be aligned with physical and temporal variables. The full set of environmental variables explains 28.3% of the variation in the species abundances, which is highly significant at *P*<0.0001 according to a permutation test [40]. The vertical first axis (CCA1) explains 31.6% of this constrained variation, and the horizontal second axis (CCA2) explains 27.3%.

**Figure 3:**
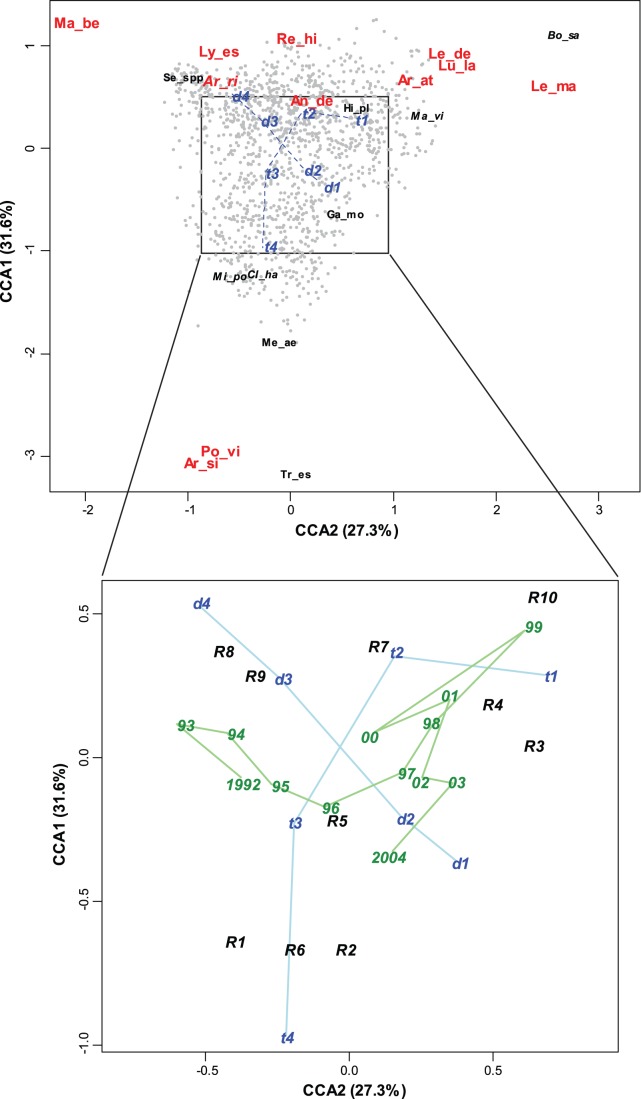
Canonical correspondence analysis (CCA) ordination biplot of axes 2 (horizontal) and axes 1 (vertical) of 55 fish taxa and 1360 stations in the period 1992−2004. (*a*) The high-contributing species are labeled in black (small font), indicator species of the three distinct fish assemblages are labeled in red (large font), cold water NE assemblage (**Ar_at** = *Artediellus atlanticus*, **Le_de** = *Leptagonus decagonus*, **Le_ma** = *Leptoclinus maculates* and **Lu_la** = *Lumpenus lampraetaeformis*), warm water S assemblage (**Ar_si** = *Argentina silus*, **Po_vi** = *Pollachius virens* and **Tr_es** = *Trisopterus esmarkii*) and deep assemblage (**Ly_es** = *Lycodes esmarkii*, **An_de** = *Anarhichas denticulatus* and **Re_hi** = *Reinhardtius hippoglossoides*) previously defined by Fossheim *et al.* [30]. Pelagic species (in italics) are associated with the northern (*Bo_sa* = *Boreogadus saida* and *Ma_vi* = *Mallotus villosus)*, southern (*Mi_po = Micromesistius poutassou* and *Cl_ha = Clupea harengus)* and deep assemblages (*Ar_ri = Arctozenus risso*). (*b*) Central section of Fig. 3a, showing the categories of bottom temperature (***t***) and depth (***d***), as in Fig. 3a, as well as those for the 10 regions (***R***) and the 13 years.

Three distinct fish assemblages, characterized by their indicator species (key species occurring in the 13 years studied (see Table 2)) and previously defined by Fossheim *et al.* [30], were identified in the CCA model (Fig. 3a) as follows:

- a warm water S assemblage (*Argentina silus*, *Pollachius virens* and *Trisopterus esmarkii*, CCA1< −3, CCA2<0),
- a cold water NE assemblage (Artediellus atlanticus., Leptagonus decagonus, Leptoclinus maculates and Lumpenus lampraetaeformis, CCA1>0.5, CCA2>1) and
- a deep assemblage (Arctozenus risso, Lycodes esmarkii, Anarhichas denticulatus and Reinhardtius hippoglossoides, CCA1>0.5, CCA2<0.5).

The species composition in the three assemblages did not change over time. Pelagic species such as *Boreogadus saida* and *Mallotus villosus* were associated with the cold water NE assemblage and *Micromesistius poutassou* and *Clupea harengus* were associated with the warm water S assemblage.

All variables in the CCA map are depicted by points representing categories; either years, regions (***R***), or ordinal categories of depth and bottom temperature (Fig. 3a, b). In the case of all variables except year, the coding is fuzzy, but the interpretation of all categories is the same. Each one of the station samples, depicted as grey dots in Fig. 3a, has a category of each variable associated with it. So each category can be displayed at the average of the station points having that particular category, using weighted averaging for the fuzzy categories and ordinary averaging for the years. Thus year 1992 appears at the lower left of the graphic because it is the average point of all the 1992 stations which must have been generally situated towards the lower left hand side (Fig. 3b). Similarly, the lowest bottom temperature category (t1) is situated at top right of the display, because it is the average of all the stations that have the lowest set of temperatures. Region 10, in the north-east of the sampling region (Fig. 1b), falls in the upper right quadrant of the display because it is the average position of all sampling points in that region (Fig. 3b). Depth turns out to be a set of points in a straight line, whereas temperature has a curved trajectory, showing that shallower samples occur in both low and high temperature regions, a fact that can be deduced from the map in Fig. 1b, which shows shallow depths in northern and southern regions. A strictly linear coding, which is the usual approach in CCA, would not be able to reflect this fact. The benefits of fuzzy coding are that nonlinear relationships with the environmental variables can be revealed, more variance can be explained in the species abundances, and the interpretation of the ordination triplots is unified because all variables are coded as categories [46].

The CCA map clearly separated the years until 1996 (CCA2<0) and the colder years after 1996 (CCA2>0) (Fig. 3). This indicates some change in the community structure, represented by CCA2 that shows change towards a community with more capelin and Polar cod from 1996 to 1999. Although years 2000 and 2004 get close to an “average” species composition the community does not seem to recover to the state of the early 90s (Fig. 3b). There was a significant difference in abundance, biomass, mean fish weight, P/D ratio and CCA2 values between the periods 1992−1997 and 1998−2004 (Student’s t-test *P*<0.01).

## Discussion

### Spatial Variation in the Fish Community

**Figure 4:**
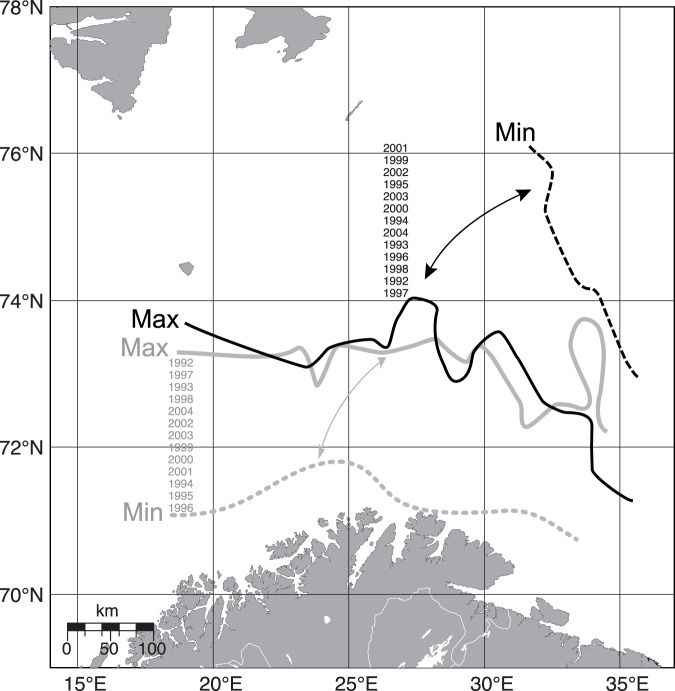
Distribution of assemblages. Maximum and minimum distribution of the cold water NE assemblage (*Artediellus atlanticus, Leptagonus decagonus*, *Leptoclinus maculates* and *Lumpenus lampraetaeformis*) in black and the warm water S assemblage (*Argentina silus*, *Pollachius virens* and *Trisopterus esmarkii*) in grey. Max indicates the widest distribution of respective assemblage (area where ≥10 individuals of the key species group have been observed over time), and Min indicates the narrowest annual distribution of both key species groups in 1992 to 2004. The ranks of years indicate the position of the maximum and the minimum distribution of the S and NE assemblages each year.

Hydrographical features such as water masses, fronts and residual currents as well as bathymetry seem to shape rather stable bottom fish assemblages in the North Atlantic [36], [47]. However, these fish assemblages may change their range of distribution along the latitude and depth gradients with an increase in temperature [48], [49]. The three fish assemblages identified in this study, a deep water assemblage, a warm water S assemblage, both associated with Atlantic water, and a cold water NE assemblage associated with mixed water (<2°C) [50], were identified in the period 1992−2004. These three assemblages have the same key species as previously identified for fish assemblages in the Barents Sea [30], and follow the same zoogeographical groupings identified in previous studies [28], [30], [51]. Hence, the species assemblages are identified over longer time periods than the 13 years studied here. Although the species may change their distribution within the 13 years studied, the S and the NE assemblages seem rather stable although they oscillate back and forth and may meet across the Barents Sea along the 2°C temperature isoline often occurring at 73–74°N (Fig. 4). Yet, the two assemblages show no consistent northwards movement as seen in neighboring North Sea fish species [25], [36]. The water mass distribution and characteristics in the Barents Sea have a major influence on the production processes [50]. Thereby the Polar Front, where Atlantic and Arctic water masses meet, and which is associated with the 200 m depth isoline in the western Barents Sea, constitutes a clear zoogeographical boundary [52]. An Arctic fish assemblage described recently by Johannesen et al. [34] and located north of the Polar Front and thereby north of our study area has many species in common with the NE assemblage.

### Temporal Change

**Figure 5:**
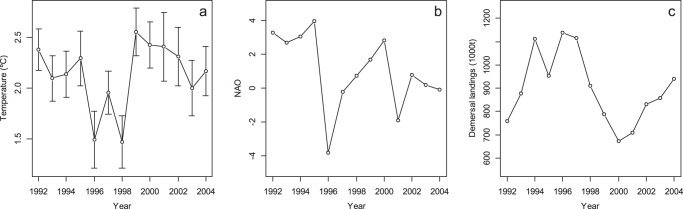
Temperature, NAO index and demersal fish landings. Mean bottom temperature (measured with Scanmar sensor attached to the survey trawl) with 95% confidence interval (*a*), the NAO index from the National Centre of Atmospheric Research, USA [80] (*b*) and demersal fish landings (*Gadus morhua*, *Melanogrammus aeglefinus*, *Pollachius virens*, *Reinhardtius hippoglossoides*, *Sebastes marinus* and *Sebastes mentella*) in the Barents Sea (ICES subareas I and II) [81] (*c*).

In the Barents Sea the winter 1996−1997 was the coldest since 1989 [53] (Fig. 5a), and resulted in a dramatic reduction in primary production in 1998 [54], high mortality of recruits, e.g. shrimp [55], and reduction in zooplankton biomass in the same period. At the same time the herring (*Clupea harengus*) stock, that migrates into the Barents Sea for food, doubled in size [56]. The feeding by herring in the pelagic layer may have resulted in less food available for demersal species and thereby a biomass reduction in the fish community (Fig. 2a). There is a potential competition between herring and cod juveniles as they have a dietary as well as a temporal and spatial overlap [57], [58], and the presence of juvenile herring reduces capelin recruitment and hence food availability in the Barents Sea [59]. In years with many capelin, these are preyed upon by cod, herring, haddock (*Melanogrammus aeglefinus*) and other demersal fish [60], such as Greenland halibut (*Reinhardtius hippoglossoides*) [61] and skates [62].

Also the high annual demersal fish landings in the early 90s had a negative effect on the fish abundance and the demersal fish abundance declined to 1/3 from 1995 to 1999 (Fig. 2e, 5d).

As in North Icelandic waters the fish biodiversity decreased after 1996 [36] (Fig. 2d). The species abundance and biomass decreased from 1996 to 1999 (Fig. 2a, 2b) and the mean fish weight declined (Fig. 2c, 2e). Thereby the community structure changed and the P/D-ratio increased as demersal fish abundance declined and pelagic species became more abundant (Fig. 2e, 2f). The low bottom temperature influenced species distribution, and opportunistic seasonal migrants, e.g., *Mallotus villosus* and *Boreogadus saida* or *Clupea harengus* and *Micromesistius poutassou* increased in cold and warm conditions respectively.

Species that change their distribution rapidly are known to have faster lifecycles and smaller body sizes than non-shifting species [25], which may explain some of the change to more pelagic species in the Barents Sea. This kind of temperature-driven change has been observed in the Northwest Atlantic [63], in the North Sea [25], in Narragansett Bay [24] and off California [64]. These studies point to climate variability as the main reason for fish community change.

The Barents Sea is a low-diversity Arctic system compared to the species-rich, temperate North Sea [65], [66]. The low species richness and diversity is a result of low sea temperature, a relation that is also found in other areas of the North Atlantic [65], [67]. Low diversity ecosystems are less resilient than high diversity ecosystems, since species loss may lead to empty niches [67]. A reduction in diversity reduces the ability of the ecosystem to compensate for change [68], and Arctic ecosystems are therefore considered to have low resilience [65]. We identified a dramatic drop in diversity from 1996 to 1999, partly driven by reduced species richness, and this is also an indication of low resilience in the fish community. Although the diversity and the abundance increased after 1999 the average fish size remains small and the pelagic species thrive (Fig. 2a–f).

### Regime Shifts in the Barents Sea?

Fish assemblages in the Barents Sea seem to have the same characteristic species over time and are determined by depth and temperature [69]–[72], but structural changes may still happen. These kinds of changes are often detected and reported when the change is obvious and a regime shift has already occurred [22]–[24]. In the Barents Sea an ecological regime shift in the 1920s was caused by warmer than normal sea temperatures, reduced sea ice coverage and enhanced Atlantic inflow [7]. Like in the 1920s, a climate change was identified in the early 1980s documented by an increase in the NAO index and temperature; however, a response in increased fish biomass as observed in the 1920s did not follow. Since the demersal fish biomass reduction in the 1960s, the large fish stocks have oscillated at a lower level [73]. This may be attributable to high fishing activity or change in oceanographic conditions, which is often observed as low primary production, or a combination of both fishing and change in climatic conditions [10].

Lees et al. [10] expected an ecological regime shift in the Barents Sea as a consequence of the climatic regime shift in 1995−1998 (decline in NAO and sea temperature), but the lack of response (decline) in the ecosystem indicators such as zooplankton biomass, gadoid recruitment and gadoid spawning stock biomass was considered to be a delay in ecological response [10]. In an ecosystem experiencing a regime shift, individual species may not respond or their response may be slow or lagged, particularly in long-lived species [6], [21], and the response by commercial species is likely to be masked by fishing mortality. We argue that change in other attributes of the ecosystem, such as fish biomass, mean individual weight, P/D ratio, and species composition, indicate significant community-wide changes in the Barents Sea, probably as a response to observed changes in bottom temperature the NAO index and fishing mortality (Fig. 5a–c). However, the time period studied here is too short to conclude that the change in fish community parameters observed in the Barents Sea between 1996 and 1999 is a more persistent (>10 year) regime shift.

Low resilience due to high fishing mortality in the mid-90s, combined with a sudden oceanographic change in 1996 seems to have resulted in an ecological change. Although the drivers, temperature, NAO index, and fishing mortality moved back to pre-change conditions, the fish community had not recovered to its previous state in 2004 (Fig. 2a–d).

### Drivers

Successful management of marine ecosystems demands a good understanding of species interactions and fisheries as well as the effect of variation in oceanographic conditions [74]. The annual catch of demersal species including cod, haddock, saithe (*Pollachius virens*) and others increased from 360 000 tons in 1990 to over 1 million tons in 1994 and stayed at this high level until 1998 [3] (Fig. 5c). The P/D ratio seems to respond with a steady increase from 1996 to 1999, mainly due to the removal of commercial demersal fish. The P/D ratio then continues to increase as recruitment of demersal fish stays low, probably due to reduced spawning stock biomass and low temperatures, and as the pelagics increase probably due to reduced predation pressure and fast lifecycles (Fig. 2e). The demersal fisheries targeting fish at a high trophic level are likely to have reduced the resilience in the Barents Sea ecosystem as seen in the NW Atlantic [65]. We hypothesize that intense fishing first reduced the resilience in the ecosystem, hence when a change in climatic conditions reduced temperatures and production, the fish community responded with a further decline in abundance and average fish weight. Yet, as fisheries and change in oceanographic conditions affect the fish community at the same time in different ways, it is not possible to separate between effects induced by the two drivers [14].

### Recovery

Although we observe a rapid decline in fish biomass, abundance and mean size and an increase in small pelagics, no obvious change in the commercial fish stocks of the Barents Sea has been observed in the study period, and the fish community may be able to recover to the composition observed in the early 1990s, characterized by relatively high abundance, high diversity and more demersal species as recruitment to large fish stocks is likely to increase in warm years [75]. However, such a re-establishment may be hindered by further increasing temperatures, as the Barents Sea is turning into a more North Sea like pelagic-dominated ecosystem [66], [76]. Forecasts predict a temperature increase that is believed to result in a primary production increase followed by a higher fish production in the Barents Sea [13], [77]–[79]. Yet, the long-term effects of warming in the Barents Sea are uncertain [50], as these studies do not evaluate the interactions between species and may therefore turn out to be too optimistic.

### Conclusions

Although the Barents Sea fish assemblages identified herein have not changed their distributions markedly, structural changes have been observed, including reduced fish biomass, abundance and mean-weight as well as increased P/D-ratio. These community characteristics did not seem to recover as the drivers, oceanographic conditions and fishing, changed back to the previous stage. A recovery may be hindered by increasing temperatures and the demersal fishery, which are transforming the Barents Sea into a more North Sea like, pelagic-dominated ecosystem.
